# The feedback related negativity encodes both social rejection and explicit social expectancy violation

**DOI:** 10.3389/fnhum.2014.00556

**Published:** 2014-07-29

**Authors:** Sai Sun, Rongjun Yu

**Affiliations:** ^1^School of Psychology and Center for Studies of Psychological Application, South China Normal UniversityGuangzhou, China; ^2^School of Economics and Management and Scientific Laboratory of Economic Behaviors, South China Normal UniversityGuangzhou, China

**Keywords:** social prediction error, social rejection, anterior cingulate cortex, FRN, event-related potential

## Abstract

Humans consistently make predictions about the valence of future events and use feedback to validate initial predictions. While the valence of outcomes provides utilitarian information, the accuracy of predictions is crucial for future performance adjustment. The feedback related negativity (FRN), identified as a marker of reward prediction error, possibly encodes social rejection and social prediction error. To test this possibility, we used event related potential (ERP) techniques combined with social tasks in which participants were required to make explicit predictions (whether others will accept their “friend request” or not, Experiment 1) or implicit predictions (whether they would like this person or not, Experiment 2) respectively, and then received social feedback. We found that the FRN is sensitive to social rejection and explicit social prediction error in Experiment 1 but not implicit social prediction error in Experiment 2. We conclude that the FRN encodes social rejection and explicit social expectancy violation.

## Introduction

Living in a sophisticated social network, humans have evolved to rely on many complex social relationships to survive. They constantly make predictions and use feedback to update initial predictions in social interactions (Brown and Brüne, 2012). The difference between actual feedback and initial prediction is termed as social prediction error or social expectancy violation (Schultz and Dickinson, 2000; Poore et al., 2012), the situation of being excluded in a social interaction is referred to as social rejection or social exclusion (Williams, 2007). For example, when you say “Good morning” to your colleague, you expect him to respond with “Good morning”. However, if he looks away and ignores your greeting, you get negative social feedback (“social rejection”) as well as social prediction error (“worse than predicted”). Such signals remind you to pay attention to the relationship with this colleague and take actions to improve your relationship with him or her. The ability to evaluate social feedback (“good or bad”) and detect social prediction errors (“expected or unexpected”) is critical for smooth and dynamic communication with others. Otherwise, undesirable social interactions may lead to mental health problems, including disruptive behavior problems, lower levels of self-esteem, higher levels of anxiety and depression (Deater-Deckard, 2001; Dodge et al., 2003; Ladd, 2006). Thus, we used event related potential (ERP) techniques in conjunction with social feedback tasks to gain a better understanding of the neural mechanisms of social expectancy violation and social exclusion.

Previous studies have demonstrated that the medial frontal cortex (MFC), especially the anterior cingulate cortex (ACC), plays a pivotal role in reward expectation and prediction error. In non-human primates, single unit recordings documented that the MFC codes for the difference between expected reward and actual outcome (prediction error) (Kennerley et al., 2011). Functional neuroimaging studies in humans demonstrated that the MFC, in particular of the ACC, is crucial for detecting discrepancies between actual and predicted outcomes, updating predictions, and making subsequent behavioral adjustments (Ridderinkhof et al., 2004; Alexander and Brown, 2010, 2011; Walsh and Anderson, 2012). A more recent electroencephalograph (EEG) study conducted by Silvetti et al. (2014) investigated both reward expectation and prediction error, and confirmed a crucial role of the MFC in rapid updating expectations through prediction errors. In specific social domain, Eisenberger et al. (2003) found that the dorsal ACC is more active during social exclusion using a “Cyberball” game. Additionally, using a social approval task, Somerville et al. (2006), investigated the brain mechanisms involved in both social prediction error and social rejection, and found that the ACC responds differentially to social expectancy violation and social exclusion.

ERP studies also identified one main component, called the feedback related negativity (FRN), which is generally believed to originate from the ACC and related to reward expectation and expectancy violation (Gehring and Willoughby, 2002; Holroyd and Coles, 2002). The FRN is a negative deflection at frontal-central recording sites and reaches maximum between 250 and 350 ms post-onset of feedback stimulus (Gehring and Willoughby, 2002). Accumulating studies revealed that the FRN is sensitive to monetary losses, erroneous feedback, and monetary prediction errors (Miltner et al., 1997; Gehring and Willoughby, 2002; Holroyd and Krigolson, 2007). In particular, the FRN is more sensitive to monetary losses compared with monetary gains, and is more negative for prediction errors than prediction congruence (Gehring and Willoughby, 2002; Holroyd and Coles, 2002; Hajcak et al., 2005, 2007). Besides FRN studies on economic decision making, a fast-growing body of experimental work has been done to investigate the FRN responses to more complex, higher-level socially relevant psychological processes, such as empathy, evaluation of fairness, social conformity, diffusion of responsibility, etc (Yu and Zhou, 2006; Boksem and De Cremer, 2010; Li et al., 2010; Kim et al., 2012).

Given the social significance of the FRN, and findings that the ACC (the main generator of the FRN) responds differently to social expectancy violation and social exclusion, it is possible that the FRN can be elicited by social prediction error and social rejection. To test this hypothesis, we used ERP techniques combined with social tasks in which participants make explicit prediction and then receive social feedback. The first goal of our study is to investigate whether social prediction error and social rejection can be encoded in the FRN.

The ability to detect subtle prediction errors is critical for smooth communication with others. Research has shown that the coupling of prediction and outcome influences the FRN. A recent ERP study found that the FRN was only associated with prediction error when subjects made predictions after rather than prior to their gambling choices (Hajcak et al., 2007), possibly because those predictions made after choices are more closely linked to outcomes. Another study found that the error related negativity (ERN) can also be elicited by errors which are not consciously detected (Shalgi and Deouell, 2012, 2013), suggesting that subliminal errors can be encoded in the ERN. Since both the ERN and the FRN are believed to be generated in the ACC, it is interesting to investigate whether the implicit prediction error can also be encoded in the FRN.

In our current research, we manipulated the connection between response and feedback by asking participants to make either explicit (Experiment 1) or implicit (Experiment 2) predictions about the outcome. In Experiment 1, participants were asked to predict whether another person would accept or reject them (that is, “Do you think this person would like to “accept or reject” your request to be his/her friend?”) and then received “accept or reject” feedback. In this situation, initial prediction and subsequent feedback are in the same “accept/reject” dimension, leading participants to build direct expectations toward the outcomes. Thus the coupling of them is direct and explicit. We expected more negative FRN components for incongruent prediction and social exclusion conditions. In Experiment 2, participants were asked to indicate whether they like or dislike another person and received “accept or reject” feedback. In general, liking another person does not necessarily mean that he/she predicts “being accepted”, but just suggests that he/she wishes to be a friend with the target person. We expected that implicit and explicit prediction error may be encoded differently by the FRN.

## Materials and methods (Experiment 1)

### Participants

Eighteen healthy volunteers (8 men, mean age ± SD, 21.83 years ± 1.72) participated in the ERP experiment. Two participants were excluded due to insufficient number of trials left after removing artifacts (less than 20, the minimal number of trials needed for a stable FRN, Pontifex et al., 2010; Marco-Pallares et al., 2011). All participants were right-handed, had normal or corrected-to-normal vision, and reported no neurological or psychiatric disorders. The study was approved by the Academic Committee of the Department of Psychology at South China Normal University. Informed consent was obtained from all participations. They were paid a uniform amount (¥40, about 7 US dollar) for their participation.

### Tasks and procedures

Participants were told that they were participating in a study about how people make first impressions based on photos on social networks (e.g., Facebook). Approximately 1 week prior to the EEG experiment, participants attended a brief informational session in the laboratory. To ensure believability, a photograph of each participant was taken, which they believed would be rated by the “evaluators” of other universities. During the EEG session, participants were required to make assessments about the so called “evaluators” faces and then received their feedback. Actually, the face stimuli were 180 digital photographs of young Chinese adult faces posing neutral expressions. These photographs either were downloaded from free Internet sources or were pictures taken of university students (with consent), which were also used in another study of our lab (Huang et al., 2014). All photos were in color and of similar quality and general appearance. The gender of pictures was matched across subjects, for male participants were presented with female pictures, and female participants were presented with male pictures.

**Figure 1:**
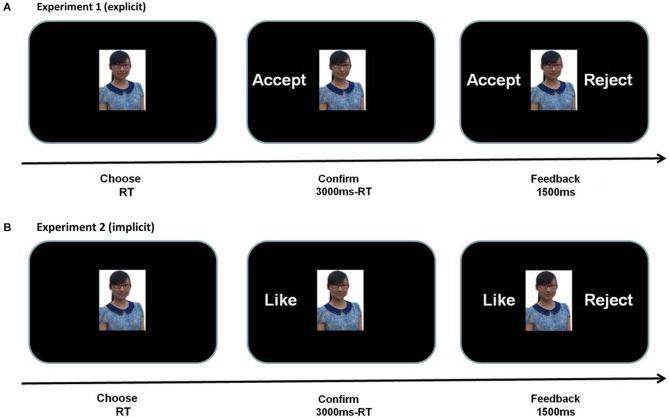
**Task design of two experiments.** For each trial, subjects viewed a target face and then responded to the question, “Do you think this person would like to “accept or reject” your friend request?” in Experiment 1 **(A)** or “Do you think you would like this person?” in Experiment 2 **(B)**. Then subject’s judgment appeared to the left of the face. After that subjects were given fictitious feedback (made up by the experimenters and believed by the subjects) indicating whether the subject was accepted or rejected by the pictured individual. The subject of the photographs gave written consent, with the approval of the Academic Committee of the Department of Psychology at South China Normal University, to the publication of their photographs. The images used in the figure are not the original images used in the study, but similar images used for illustrative purposes only.

At the beginning of each trial, an image of face was presented in the center of the screen (3.5° high, 4.5° wide in visual angle, white against a black background) and participants were required to predict whether the person appearing on the screen would like to accept (left button) his/her request to be his/her friend or not (right button) within 3000 ms (Figure 1A). Then the “accept” or “reject” prediction was presented on the left side of the screen. After that, participants received “accept” or “reject” feedback for 1500 ms indicating how the “evaluators” of other universities had previously rated them, from which participants also learned whether their initial prediction was consistent with the following feedback or not. Unbeknown to participants, following either “accept” prediction or “reject” prediction, the probability of “accept” or “reject” feedback was presented at 50% chance level. The association between face stimuli and types of feedback was randomized and the order of the four types of feedback was also randomized. The whole experiment consisted of two blocks of 90 trials each.

At the end of the EEG experiment, participants were asked to indicate how satisfied and surprised they felt for each type of feedback (i.e., predicting to be accepted and actually being accepted; predicting to be rejected and actually being accepted; predicting to be rejected and actually being rejected; predicting to be accepted and actually being rejected. see below for details) using a 10-point Likert scale respectively (1 = not at all, 10 = very intensely). The Rejection Sensitivity Questionnaire (RSQ), which measures an individual’s level of rejection sensitivity, was also used. Surveys showed that the internal consistency (alpha) and test-retest reliability of RSQ are high, being 0.81 and 0.83 respectively (Downey and Feldman, 1996).

### ERP recording and processing

The participant was seated comfortably about 1.5 m away from the computer screen in a dimly lit and electromagnetically shielded room. The experiment was administered on a Lenovo computer in CRT display, with 1024*768 resolutions, using E-prime (Psychology Software Tools, Inc. Pittsburgh, PA, USA^1^) software to control the presentation and timing of stimuli. The EEG was recorded from 64 scalp sites using tin electrodes mounted in an elastic cap (Neuroscan4.5) according to the International 10–20 system. The vertical-oculogram (VEOG) was recorded from left supra-orbital and infra-orbital electrodes. The horizontal electro-oculogram (HEOG) was recorded from electrodes placed 1.5 cm lateral to left and right external mastoid. All electrode recordings were referenced to an electrode placed on the left mastoid, and the impedance was maintained below 5 KΩ. The EEG and electro-oculogram (EOG) were amplified using a 0.05–70 Hz band-pass and were continuously sampled at 500 Hz/channel for off-line analysis. The EEG data were re-referenced off-line to linked mastoid electrodes by subtracting from each sample of data recorded at each channel one-half the activity recorded at the right mastoid. Ocular artifacts were corrected with an eye-movement correction algorithm (Gratton et al., 1983). Epochs of 800 ms (with 200 ms pre-stimulus baseline) EEG for each electrode were time-locked to the onset of feedback stimuli and were sorted by experimental conditions. Then the data were baseline corrected by subtracting from the average activity of that channel during the baseline period. The FRN data were filtered using a 1–20 Hz band-pass (24 dB octave roll off) to remove low-frequency waves from the EEG. All trials in which EEG voltages exceeded a threshold of ±70 μ*ν* during the recording epoch were excluded from analysis.

### ERP analysis

The average FRN amplitudes were measured in a window of 300–400 ms after the onset of feedback. Previous studies also used mean amplitude measure for FRN (Zhou et al., 2010; Yu et al., 2011). We focused on the FRN responses at the anterior frontal midline electrodes (Fz), since the FRN was the largest at this electrode. The FRN data were entered into ANOVAs, with expectancy conditions (congruent vs. incongruent) and feedbacks (accepted vs. rejected) being two within subject factors (The four conditions are as follows: AA: predicting being accepted and actually being accepted; RA: predicting being rejected and actually being accepted; RR: predicting being rejected and actually being rejected; AR: predicting being accepted and actually being rejected). Thus, AA and RR are congruent condition, and RA and AR are incongruent condition; AA and RA are “accepted” feedback, and RR and AR are “rejected” feedback. The Mauchly test was used to assess the validity of the sphericity assumption in all ANOVAs. Greenhouse-Geisser corrections were used when sphericity was violated. Alpha level for all tests was 0.05 and two-tailed.

## Results (Experiment 1)

### Behavioral results

**Figure 2:**
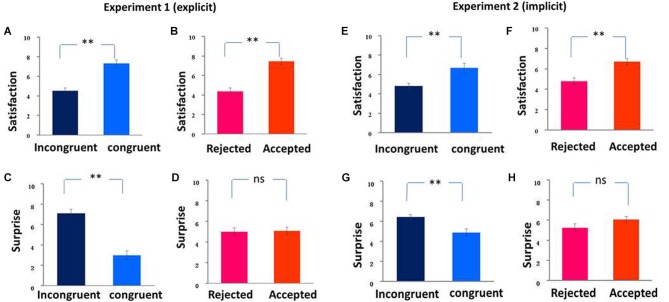
**Post-experiment subjective ratings of two experiments during social feedback period.** The self-reporting satisfaction and surprise scores (mean ± SE) was shown in (**A–H**). Participants felt more satisfied for congruent than incongruent feedback **(A)**, and for accepted than rejected feedback **(B)**. Participants felt more surprised by congruent than incongruent feedback **(C)** but no significant difference between accepted and rejected feedback **(D)**. We found similar behavioral results in Experiment 2 (**E**–**H**). ** *p* < 0.01, * *p* < 0.05.

Participants were more likely to predict being accepted (mean ± SD, 59% ± 10.28%, range from 33% to 75%) than to predict being rejected, *t*_(15)_ = 3.48, *p* = 0.003, suggesting that people are generally optimistic. For the sake of completeness, we also reported the results of reaction time. The RTs did not differ between the two conditions (mean ± SD, 951 ms ± 169.35 for predict being accepted vs. 968 ms ± 181.49 for predict being rejected), *t*_(15)_ < 1, suggesting that equal efforts were involved in initial prediction response. For the self-reported satisfaction, ANOVA analysis revealed a significant main effect of expectancy violation, *F*_(1, 15)_ = 26.91, *p* < 0.001. Specifically, participants felt more satisfied when their prediction was consistent (mean ± SD, 7.31 ± 1.37) than inconsistent with the feedbacks (mean ± SD, 4.53 ± 1.12; see Figure 2A). There was also a significant main effect of social feedback, *F*_(1, 15)_ = 27.81, *p* < 0.001. Participants felt more satisfied for being accepted (mean ± SD, 7.47 ± 1.27) than for being rejected (mean ± SD, 4.38 ± 1.41; see Figure 2B). No significant interactions were found, *F*_(1, 15)_ < 1.

For self-reported surprise, we also found a significant main effect of expectancy violation, *F*_(1, 15)_ = 66.13, *p* < 0.001. Participants felt more surprised when receiving incongruent (mean ± SD, 7.09 ± 1.57) than congruent feedback (mean ± SD, 2.97 ± 1.77; see Figure 2C). No significant main effect of social feedback was found, *F*_(1, 15)_ < 1 (see Figure 2D). The interaction between the two factors was marginally significant, *F*_(1, 15)_ =3.15, *p* = 0.096.

### ERP results

**Figure 3:**
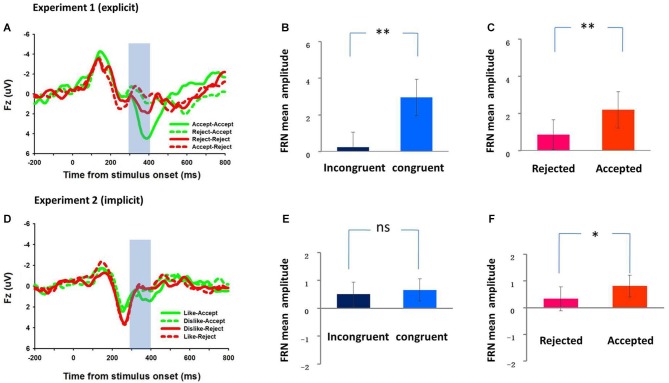
**The ERP grand-average waveforms during the social feedback period, and the mean amplitudes of the FRN elicited by expectancy violation (incongruent vs. congruent) and social valence (rejected vs. accepted) of two experiments**. The ERP grand average waveforms at channel Fz for four conditions in both Experiment 1 and Experiment 2 were shown in (**A** and **D**). The shaded 300–400 ms time window was for the calculation of average amplitudes of FRN effects. The mean amplitudes of the FRN (mean ± SE) were shown in the right panel. In Experiment 1, the FRN is more negative for expectancy violation than for expectancy congruence **(B)**, and more negative for being rejected than for being accepted **(C)**. In Experiment 2, the main effect of expectancy violation on the FRN was not significant **(E)** while the FRN is more negative for being rejected than for being accepted **(F)**. ** *p* < 0.01, * *p* < 0.05.

Figure 3 depicts the grand average waveforms of four experimental conditions at Fz (see Figure 3A). The mean FRN amplitudes in the 300–400 ms window were submitted to a 2 by 2 repeated measures ANOVA, with expectancy conditions (congruent vs. incongruent) and actual feedbacks (rejected vs. accepted) being independent factors. The average number of trials in four various categories was 50 ± 11.91 (mean ± SD, AA), 38 ± 10.09 (mean ± SD, RA), 36 ± 10.03 (mean ± SD, RR) and 52 ± 9.06 (mean ± SD, AR). The average FRN amplitudes revealed a significant main effect of feedback, *F*_(1, 15)_ = 6.82, *p* = 0.002, with a more negative FRN for “reject” than for “accept” feedback (mean ± SD, 0.86 μ*ν* ± 3.21 vs. 2.19 μ*ν* ± 3.92, respectively) and a significant main effect of expectancy congruence, *F*_(1, 15)_ = 28.31, *p* < 0.001, with a more negative FRN for expectancy violation than for expectancy congruence (mean ± SD, 0.24 μ*ν* ± 3.25 vs. 2.95 μ*ν* ± 3.96, respectively; see Figures 3B,C). Results suggested that the FRN was sensitive to both explicit social expectancy violation and explicit social rejection. We found no significant interaction effects between the two factors, *F*_(1, 15)_ = 2.50, *p* < 0.135.

Additionally, we also analyzed the FRN effects using peak-to-peak (N2 minus P2) amplitude measure on the Fz electrode (Hajcak et al., 2006; Holroyd et al., 2006). The N2 was defined as the most negative peak in the time window between 380 and 480 ms, and P2 was defined as the largest positive peak in the time window from 200 to 300 ms. ANOVA analysis revealed a similar pattern of FRN effects with mean amplitude measures. Specifically, we found a significant main effect of social feedback, *F*_(1, 15)_ = 4.99, *p* = 0.041, a significant main effect of expectancy violation, *F*_(1, 15)_ =14.08, *p* = 0.002, and no significant interaction effect, *F*_(1, 15)_ = 1.09, *p* = 0.314.

For the time window used for detecting the FRN (300–400 ms), it was possible that the variance of FRN amplitudes may be overlapped with the early P2 (200–300 ms) or later P3a (380–480 ms). To minimize the influence of P2 and P3a on the measure of FRN, we also measured the FRN magnitude of the different waveforms. A similar pattern of effects was observed. One sample *t*-test revealed a significant effect on explicit expectancy violation [*t*_(15)_ = 4.528, *p* < 0.001] and social rejection [*t*_(15)_ = 2.631, *p* = 0.019]. Thus, our findings were not affected by P2 and P3a activity.

**Figure 4:**
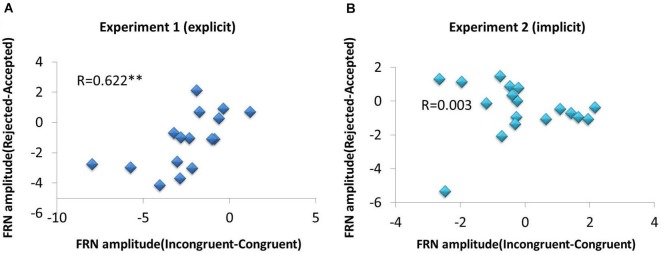
**Scatter plots of the association between the expectancy violation effect on the FRN and the social rejection effect on the FRN.** The expectancy violation effect on the FRN (incongruent minus congruent) has a significant positive correlation with the social rejection effect on the FRN (rejected minus accepted) in Experiment 1 **(A)** but not in Experiment 2 **(B)**. ** *p* < 0.01, * *p* < 0.05.

The expectancy violation effect on the FRN (incongruent minus congruent) was significantly correlated with the social rejection effect on the FRN (rejected minus accepted), *r* = 0.622, *p* = 0.01, suggesting that social prediction and outcome evaluation may share a common neural mechanism (see Figure 4A). Additionally, we also found a significant correlation between self-report satisfaction and rejection sensitivity scale, *r* = −0.675, *p* = 0.004, and a significant correlation between self-report surprise and rejection sensitivity scale, *r* = 0.612, *p* = 0.012. These behavioral correlations suggested that the more sensitive to social rejection, the more unsatisfactory or surprised individuals may feel. Given the small sample size, we also applied the bootstrapping method to conduct bivariate correlation analysis with M-Plus software. The bootstrapping analysis yielded similar results.

## Discussion

At the behavioral level, participants were generally optimistic about their chances of being accepted (59% prediction of being accepted). Our study also demonstrated behavioral differences in self-reported satisfaction ratings. When receiving negative feedback (i.e., Rejected), they felt more dissatisfied but not more surprised, suggesting that satisfaction and surprise are two independent measures. When the feedback was deviated from the initial prediction, regardless of whether the feedback is positive or negative, they felt more dissatisfied and more surprised, suggesting that the social expectancy violation is not only surprising but also unpleasant. These behavioral results showed that our experimental manipulation successfully influenced the participants’ subjective emotions.

At the neural level, we have identified a more negative FRN waveform when people were rejected and their explicit expectancy was violated. Our ERP findings are in agreement with studies by Kim et al. (2012) and Boksem and De Cremer (2010), in which the FRN is more pronounced for social norm violations and unfair offers (similar to social prediction error and social rejection). Our tentative researches provide further neurophysiological evidence that the FRN can encode both explicit prediction error and explicit rejection in social domain. However, we found no significant interaction effect, suggesting that the FRN encodes expectancy violation independently, without taking the valence of outcomes into account. Our results were consistent with the response-outcome (PRO) theory, which suggests that the key function of ACC is to predict the possible outcomes of actions and signaling unexpectedness of events, regardless of the valence of outcomes (Alexander and Brown, 2010, 2011). More studies are needed to examine whether this effect we found exists only in the current setup, or if it can be generalized to other social prediction tasks.

In our study, we also found a significant correlation between the FRN difference amplitude of expectancy violation (incongruent minus congruent) and the FRN difference amplitude of social rejection (rejected minus accepted). The results suggest that social prediction error and social rejection may share a common neural mechanism. Whether social expectancy violation and social rejection are integrated in the same region (e.g., the ACC), or instead segregated into subdivisions, is currently under debate. Devinsky et al. (1995) and Bush et al. (2000) analyzed a broad range of functional imaging, electrophysiological studies, and argued that the “rostral” ACC is specialized for affective processes, whereas the “dorsal” ACC is specialized for cognitive processes. Shackman et al. (2011) reviewed previous work and suggested that negative emotion, pain and cognitive control are anatomically and functionally integrated at the subdivision in the cingulate cortex. Specifically, Somerville et al. (2006) demonstrated that the dACC is sensitive to expectancy violations, whereas the ventral ACC (vACC) is responsive to social feedback. Their findings support a general role for the dACC in the processing of cognitive conflicts and a more specific role for the vACC in affective evaluations. Though they are not in the exact same brain region of the ACC, we still postulate that the FRN activity elicited by social prediction error and social rejection could be encoded in a similar profile, which may be allocated to the ACC, but involving in different parts.

To conclude, both explicit social prediction error and explicit social rejection can be encoded by the FRN. One remaining question is whether or not the violation of implicit prediction can also elicit the FRN. To explore this question, we conducted another experiment, in which participants were required to subjectively judge whether they would like another person or not and then received the social feedback. In general, liking another person does not necessarily mean that one predicts another person would accept them but only suggests that one wishes to be a friend with the target person. Thus, the connection is indirect and implicit between “like or dislike” responses and the subsequent feedback, which can be considered as an implicit prediction. We hypothesized that the implicit prediction error can be encoded differently from the explicit one.

## Materials and methods (Experiment 2)

### Participants

Twenty-four healthy volunteers (11 men, mean age ± SD, 22.13 ± 1.56 years) participated in the ERP experiment. Six participants were excluded from the group analysis, because two of them did not believe in our experimental manipulation, and the other were excluded for insufficient number of trials left after removing artifacts (less than twenty, the minimal number of trials needed for a stable FRN, Pontifex et al., 2010; Marco-Pallares et al., 2011). All participants were right-handed, had normal or corrected-to-normal vision, and reported no neurological or psychiatric disorders. The study was approved by the Academic Committee of the Department of Psychology at South China Normal University. Informed consent was obtained from all participations. They were paid a uniform amount (¥40, about 7 US dollar) for their participation.

### Tasks and procedures

The manipulation of Experiment 2 was identical to Experiment 1 except that the initial explicit prediction of “accept” or “reject” was replaced by subjective choices of “like” or “dislike”. To confirm that making like/dislike choices indeed builds expectation toward the outcome (accept/reject), we did another behavioral study in which participants were required to make “like or dislike” choices toward each photo in session 1 and then to predict “being accepted or rejected” in session 2. Correlation analysis revealed a significant positive correlation between explicit prediction (i.e., accept/reject) and subjective judgment (i.e., like/dislike), *p* < 0.001, suggesting an association between them (*r* = 0.227). The following feedback is as same as Experiment 1. At the end of experiment, participants were required to subjectively rate their satisfaction and surprise degree toward four different conditions.

### ERP analysis

The ERP analysis of Experiment 2 was identical to Experiment 1. The average FRN data were entered into ANOVAs, with implicit expectancy conditions (consistent vs. inconsistent) and feedback (accepted vs. rejected) being two within subject factors (The four conditions are as follows. LA: choosing like and actually being accepted; DA: choosing dislike and actually being accepted; DR: choosing dislike and actually being rejected; LR: choosing like and actually being rejected).

## Results (Experiment 2)

### Behavioral results

Participants tend to respond with more “dislikes” (mean ± SD, 56% ± 12.66%, range from 34% to 73%) than “likes”, and there was a marginal significant difference between them, *t*_(17)_ = 1.89, *p* = 0.075. For the sake of completeness, we also reported the results of reaction time. Results show that the RTs differ slightly between two conditions (mean ± SD, 961 ms ± 184.32 to choose dislike vs. 1000 ms ± 164.61 to choose like), *t*_(17)_ = −1.84, *p* = 0.083, suggesting that making “disliking” requires more effort than making “liking”.

As respect to the self-reported satisfaction, ANOVAs results revealed a significant main effect of expectancy violation, *F*_(1, 17)_ = 8.91, *p* = 0.008. Specifically, participants felt more satisfied when their prediction was consistent (mean ± SD, 6.67 ± 2.00) rather than inconsistent with the feedback (mean ± SD, 4.81 ± 1.26; see Figure 2E). There was also a significant main effect of social feedback, *F*_(1, 17)_ = 19.01, *p* < 0.001. Participants felt more satisfied for being accepted (mean ± SD, 6.69 ± 1.37) than for being rejected (mean ± SD, 4.78 ± 1.39; see Figure 2F). No significant interaction was observed, *F*_(1, 17)_ < 1.

For self-reported surprise, we found a significant main effect of expectancy violation, *F*_(1, 17)_ = 11.21, *p* = 0.004. Participants felt more surprised when receiving incongruent (mean ± SD, 6.42 ± 1.09) than congruent feedback (mean ± SD, 4.89 ± 1.48; see Figure 2G). No significant main effect of social feedback (see Figure 2H), and interaction effect between the two factors was found, *F*_(1, 17)_ = 2.95, *p* = 0.172; *F*_(1, 17)_ < 1.

### ERP results

Figure 3 depicts grand average waveforms for four conditions at Fz (see Figure 3D). The mean FRN amplitudes in the 300–400 ms window were submitted to a 2 by 2 repeated-measures ANOVA, with implicit expectancy conditions (consistent vs. inconsistent) and actual feedbacks (rejected vs. accepted) as independent factors. The average number of trials in four various categories was 37 ± 10.66 (mean ± SD, LA), 51 ± 11.22 (mean ± SD, DA), 47 ± 12.68 (mean ± SD, DR), and 41 ± 12.25 (mean ± SD, LR). The average FRN amplitudes revealed a main effect of feedback, *F*_(1, 17)_ = 7.87, *p* = 0.012, with a larger FRN for “reject” than for “accept” feedback (mean ± SD, 0.33 μ*ν* ± 0.189 vs. 0.81 μ*ν* ± 1.73, respectively) suggesting that the FRN was sensitive to social rejection (see Figure 3F). No significant main effect of prediction (*F*_(1, 17)_ < 1; see Figure 3E) or interaction effect (*F*_(1, 17)_ = 2.08, *p* = 0.167) was found in FRN amplitude, indicating that the FRN was not sensitive to implicit prediction error. No significant correlation on the FRN between congruence effect (incongruent minus congruent) and social valence effect (rejected minus accepted) was found, *r* = 0.003, *p* = 0.992 (see Figure 4B). No other significant behavioral correlations were found.

### General discussion

In both experiments, participants felt more dissatisfied when receiving negative social feedback. They also felt more dissatisfied and surprised when their predictions (explicit or implicit) were violated. At the neural level, we found that both negative feedback and social prediction error elicited the FRN when there is a close link between prediction and outcome (explicit prediction error in Experiment 1), but not when the prediction is only implicitly linked with outcome (implicit prediction error in Experiment 2). One previous study demonstrated that the FRN is sensitive to reward prediction violations, but it depends on the close coupling of prediction and outcome. If prediction is not followed by an immediate feedback, the FRN was insensitive to reward prediction violations (Hajcak et al., 2007). Similarly, in Experiment 2, participants were asked to indicate whether they “like or dislike” another person and received “accept or reject” feedback. However, “liking” another person does not always mean that he/she predicts the person would accept them but only suggests that one wishes to be a friend with the target person. The implicit prediction error cannot be encoded by the FRN for their weak response-outcome associations. Thus, our brain mainly detect signals involved with negative feedback and explicit social prediction error, which helps us validate initial predictions and make behavioral adjustments in future social interactions.

It is possible that the FRN effect can be elicited by visual differences between two words (e.g., accept and reject). A previous study has revealed that the perceptual conflict of Chinese characters could enlarge the FRN waveform and activate the ACC more strongly (Jia et al., 2007). Here, we compared the FRN amplitude between AA and RR conditions, and found a significant difference between them, *p* = 0.004. Since two identical words were presented in the above two conditions (i.e., “accept and accept” or “reject and reject”), there would be no significant difference in the FRN according to the visual difference explanation. Thus, our findings cannot simply be explained by the mismatch between two Chinese characters in the feedback stage.

Several important limitations in this study are worth mentioning. First, though the ACC is generally believed to be the main generator of the FRN, no specific evidence in our study has been provided to link the FRN amplitude with the ACC activity. Other neuroimaging methods with high spatial resolution (e.g., fMRI) are needed to locate the source of the FRN more precisely. Additionally, for the correlation between social prediction error and social rejection on FRN, some trials were involved in both the rejection minus accepted contrast and the incongruent minus congruent contrast, which may induce an artificial correlation between them. Thus, our correlation results need to be interpreted with caution. Finally, there is no direct evidence to prove that all participants were building implicit expectations throughout the entire task. Our preliminary results do find a positive correlation between subjective “like/dislike” judgment and explicit “accept/reject” prediction, *p* < 0.001. However, the effect size (*r* = 0.227) for the correlation is small and it only gives indirect support to the link between making “like/dislike’ judgment and building implicit expectations on acceptation/rejection. Our results therefore need to be interpreted with caution.

In summary, we found that the FRN encodes both social rejection and explicit social prediction errors. The FRN may function as a general mechanism that evaluates whether the outcome is positive or negative, whether the feedback is consistent or inconsistent with initial prediction in social domains. Individuals may learn from explicit prediction errors and social feedback to evaluate social relationships and adjust themselves to future social interactions. These findings, if being replicated, can enhance our understanding of the functional significance of the FRN in social domains (e.g., the ACC).

## Author contributions

Rongjun Yu conceived and designed the experiments. Sai Sun performed the experiments. Sai Sun analyzed the data. Sai Sun and Rongjun Yu wrote the main manuscript. All authors read and approved the final manuscript.

## Conflict of interest statement

The authors declare that the research was conducted in the absence of any commercial or financial relationships that could be construed as a potential conflict of interest.

## Footnotes

^1^www.pstnet.com/eprime
